# Multistep metabolic engineered *Klebsiella oxytoca* for efficient l-leucine production

**DOI:** 10.1016/j.synbio.2026.01.024

**Published:** 2026-02-06

**Authors:** Weikang Sun, Qiaoyue Yang, Shuo Wang, Lingru Gong, Zhi Zhou, Mingyuan Liu, Xiaoxu Tan, Qianjin Kang, Wensi Meng, Yidong Liu, Zhaoqi Kang, Ping Xu, Cuiqing Ma, Chao Gao, Chuanjuan Lü

**Affiliations:** a State Key Laboratory of Microbial Technology, Shandong University, Qingdao, China; b Zibo Integrated Traditional Chinese and Western Medicine Hospital, Zibo, China; c State Key Laboratory of Microbial Metabolism, Shanghai Jiao Tong University, Shanghai, China

**Keywords:** l-Leucine, *Klebsiella oxytoca*, Metabolic engineering, l-Valine, Metabolic flux redirection

## Abstract

l-Leucine is widely applied in food, feed and medical industries. In this work, an efficient l-leucine producing strain *Klebsiella oxytoca* LKO-14 was constructed based on an l-valine producer *K*. *oxytoca* VKO-9. The exogenous l-leucine biosynthesis pathway was introduced to achieve l-leucine accumulation and decrease l-valine production. Modifying l-leucine transport system and optimizing copy numbers of genes including *CgleuA*^*M*^ encoding l-leucine insensitive isopropylmalate synthase, *EcleuCD* encoding isopropylmalate isomerase, *EcleuB* encoding isopropylmalate dehydrogenase, *NopheDH* encoding phenylalanine dehydrogenase, were conducted to increase l-leucine production. The expression of *budB* encoding α-acetolactate synthase and *EcilvD* encoding dihydroxyacid dehydratase were also enhanced to improve precursor supply of l-leucine. In addition, the dihydroxy acid dehydratase from *Streptococcus mutans* containing an oxygen-tolerant [2Fe–2S] cluster was introduced to further enhance l-leucine production. Finally, the plasmid free and inducer independent *K. oxytoca* LKO-14 produced 70.1 g/L l-leucine, with a yield of 0.347 g/g and a productivity of 1.46 g/L/h, respectively.

## Graphical abstract

**Figure:**
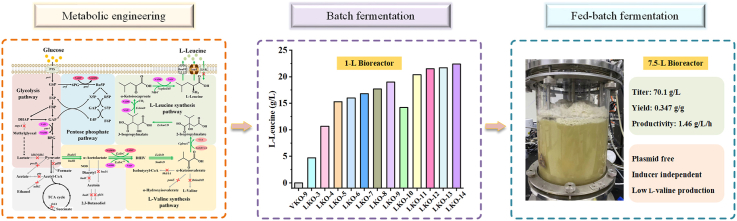

## 1 Introduction

l-Leucine is an important branched-chain amino acid with diverse applications and broad market demand [[1], [2], [3], [4], [5]]. The vast majority of industrial generated l-leucine has been applied as the feed additive [6]. Its addition can improve the disease resistance and growth performance of poultry, swine and aquatic animals [[7], [8], [9], [10]]. Nowadays, the industrial production of l-leucine predominantly relies on microbial fermentation [11]. *Escherichia coli* and *Corynebacterium glutamicum* are the two main microorganisms employed for l-leucine fermentation [[12], [13], [14], [15], [16]]. The highest concentrations of l-leucine produced by *C. glutamicum* and *E. coli* were 54.3 g/L and 63.29 g/L, respectively [12,17].

The biosynthesis of l-leucine begins with the condensation of 2-ketoisovalerate with acetyl-CoA, and proceeds via the catalytic actions of isopropylmalate synthase (IPMS), isopropylmalate isomerase (IPMI), and isopropylmalate dehydrogenase (IPMDH) to form 2-ketoisocaproate. Then, 2-ketoisocaproate can be transformed into l-leucine by branched-chain amino acid transaminase or leucine dehydrogenase (LeuDH) [18]. 2-Ketoisovalerate, the vital precursor for l-leucine production, is generated from pyruvate by the action of α-acetohydroxyacid synthase (AHAS), acetohydroxyacid isomeroreductase (AHAIR), and dihydroxyacid dehydratase (DHAD). Importantly, AHAS contains a regulatory subunit and is feedback inhibited by l-leucine and l-valine [16].

*Klebsiella oxytoca* is a Risk Group 2 organism with rapid growth speed and wide substrate spectrum. It has been utilized for various bio-based products generation [[19], [20], [21]]. Besides AHAS, *K. oxytoca* expresses α-acetolactate synthase (BudB), another enzyme catalyzing the condensation of pyruvate to produce α-acetolactate. BudB is responsible for 2,3-butanediol production in *K. oxytoca* and lacks a regulatory subunit [22]. Thus, it is not feedback inhibited by l-leucine and l-valine. The insensitivity characteristic of BudB makes *K. oxytoca* a promising candidate for l-valine and l-leucine production. Recently, Cao et al. obtained a recombinant *K. oxytoca* strain VKO-9 through rearranging the metabolic flux from 2,3-butanediol manufacture to l-valine generation [23]. *K. oxytoca* VKO-9 can produce up to 122 g/L l-valine, which is higher than those obtained with *E*. *coli* and *C*. *glutamicum*.

**Fig. 1:**
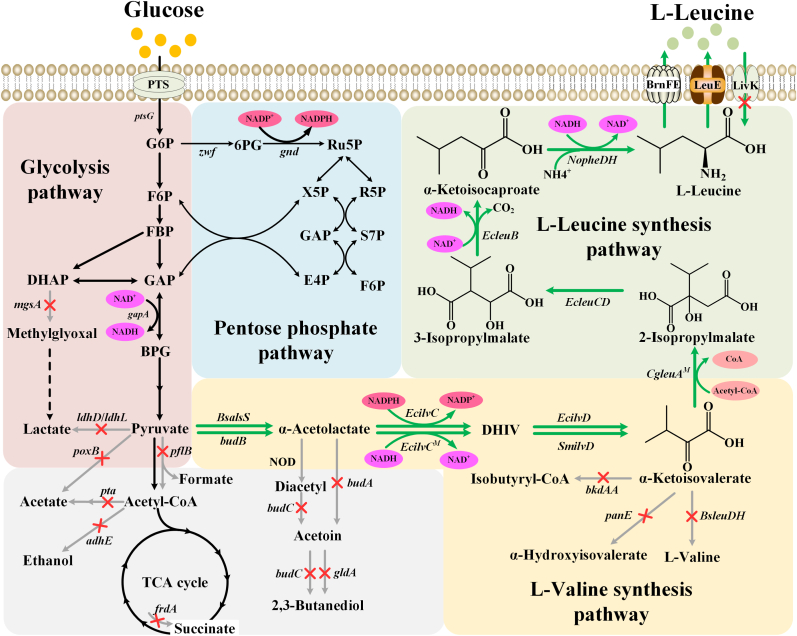
Metabolic engineering of l-valine-producing strain *K. oxytoca* VKO-9 for l-leucine production. Red crosses indicated blocked pathways and green arrows indicate overexpressed pathways in the metabolic engineered strain. PTS, phosphotransferase system; TCA cycle, tricarboxylic acid cycle; NOD, non-enzymatic oxidative decarboxylation; G6P, glucose-6-phosphate; F6P, fructose-6-phosphate; FBP, fructose-1,6-bisphosphate; GAP, glyceraldehyde-3-phosphate; DHAP, dihydroxyacetone phosphate; BPG, 1,3-bisphosphoglycerate; 6PG, 6-phosphogluconate; Ru5P, ribulose-5-phosphate; X5P, xylulose-5-phosphate; R5P, ribose-5-phosphate; S7P, sedoheptulose-7-phosphate; E4P, erythrose-4-phosphate; DHIV, 2,3-dihydroxyisovalerate; *ptsG*, glucose-specific phosphotransferase system IIBC component encoding gene; *zwf*, glucose-6-phosphate dehydrogenase encoding gene; *gnd*, 6-phosphategluconate dehydrogenase encoding gene; *gapA*, glyceraldehyde-3-phosphate dehydrogenase encoding gene; *mgsA*, methylglyoxal synthase encoding gene; *ldhD*, d-lactate dehydrogenase encoding gene; *ldhL*, l-lactate dehydrogenase encoding gene; *poxB*, pyruvate oxidase encoding gene; *pflB*, pyruvate formate-lyase encoding gene; *pta*, phosphate acetyltransferase encoding gene; *adhE*, alcohol dehydrogenase encoding gene; *frdA*, α-subunit of fumarate reductase encoding gene; *budB*, α-acetolactate synthase encoding gene; *BsalsS*, α-acetolactate synthase encoding gene from *Bacillus subtilis* 168; *budA*, α-acetolactate decarboxylase encoding gene; *budC*, *meso*-2,3-butanediol dehydrogenase encoding gene; *gldA*, glycerol dehydrogenase encoding gene; *bkdAA*, α-subunit of branched-chain keto acid dehydrogenase complex encoding gene; *BsleuDH*, leucine dehydrogenase encoding gene from *B. subtilis* 168; *EcilvC*, acetohydroxyacid isomeroreductase encoding gene from *E. coli* W3110; *EcilvC*^*M*^, mutant acetohydroxyacid isomeroreductase (L67E, R68F, and K75E) encoding gene from *E. coli* W3110; *EcilvD*, dihydroxyacid dehydratase encoding gene from *E. coli* W3110; *SmilvD*, dihydroxyacid dehydratase encoding gene from *Streptococcus mutans*; *panE*, α-hydroxyacid dehydrogenase encoding gene; *CgleuA*^*M*^, mutant isopropylmalate synthase (R529H and G532D) encoding gene from *C. glutamicum* ATCC13032; *EcleuCD*, 2-isopropylmalate isomerase encoding gene from *E. coli* W3110; *EcleuB*, isopropylmalate dehydrogenase encoding gene from *E. coli* W3110; *NopheDH*, phenylalanine dehydrogenase encoding gene from *Nocardia* sp. strain 239; BrnFE, branched-chain amino acid transporter from *C. glutamicum* ATCC13869; LeuE, l-leucine exporter; LivK, l-leucine importer.

In this study, *K. oxytoca* VKO-9 was further metabolically engineered for efficient l-leucine synthesis. A recombinant strain *K. oxytoca* LKO-14 was constructed via overexpressing key enzymes for l-leucine production, blocking branch pathways, rebalancing of cofactor, and modifying l-leucine transport system (Fig. 1). l-Leucine at a high concentration of 70.1 g/L was manufactured by *K. oxytoca* LKO-14, with a yield of 0.347 g/g and a productivity of 1.46 g/L/h. Importantly, the key genes for l-leucine synthesis were integrated at genome of *K. oxytoca* LKO-14 and controlled under constitutive promoters. *K. oxytoca* LKO-14 may be a promising alternative for stable industrial l-leucine production.

## 2 Materials and methods

### 2.1 Strains, plasmids, and medium

The strains and plasmids used in this study are listed in Table S1. *E. coli* and *K. oxytoca* culture were carried out in LB medium for strain construction and seed preparation. Spectinomycin (50 mg/L), chloramphenicol (40 mg/L), and kanamycin (50 mg/L) were added as appropriate. The fermentation medium (1 L) for l-leucine production contained 60 g glucose, 2 g KH_2_PO_4_, 10 g K_2_HPO_4_·3H_2_O, 0.1 g MgSO_4_·7H_2_O, 5 g yeast extract, and 10 g (NH_4_)_2_SO_4_, and 1 mL metal ion stock solution [24].

### *2.2* *Genetic engineering of Klebsiella oxytoca*

Genetic engineering at genome of *K*. *oxytoca* were performed using pEcCas_Cm_ carrying the Cas9 nuclease and pEcgRNA carrying the guide RNA (gRNA) [25]. Primers gRNA-*BsleuDH*-1 and gRNA-*BsleuDH*-2 were annealed to generate double-stranded DNA targeting *BsleuDH* (Table S2). The double-stranded DNA was ligated to pEcgRNA plasmid to construct pEcgRNA-Δ*BsleuDH*. Primers Δ*BsleuDH*::*NopheDH*-1/Δ*BsleuDH*::*NopheDH*-2 and Δ*BsleuDH*::*NopheDH*-5/Δ*BsleuDH*::*NopheDH*-6 were used to amplify upstream and downstream homologous arms of *BsleuDH* from *K. oxytoca* VKO-9 genome, while primers Δ*BsleuDH*::*NopheDH*-3/Δ*BsleuDH*::*NopheDH*-4 were used to amplify *NopheDH* from plasmid pET28a-*NopheDH*. The three fragments were assembled to generate the donor DNA, which was then co-transformed with plasmid pEcgRNA-Δ*BsleuDH* into *K. oxytoca* harboring plasmid pEcCas_Cm_ via electroporation. Positive clones with replacement of *BsleuDH* by *NopheDH* were selected on plates with 40 μg/mL chloramphenicol and 50 μg/mL spectinomycin. Elimination of plasmids pEcgRNA and pEcCas_Cm_ was carried out as described previously [24].

### 2.3 Protein expression and purification

The recombinant plasmids pET28a-*BsleuDH* and pET28a-*NopheDH* were transformed into *E. coli* BL21 (DE3) to obtain expression strains for leucine dehydrogenase (LeuDH) and phenylalanine dehydrogenase (PheDH), respectively. Expression of the target proteins was induced at 16 °C and 160 rpm for 12 h. The collected cells were resuspended in binding buffer (20 mM sodium phosphate, 500 mM NaCl, and 20 mM imidazole, pH 7.4) and then lysed using a high pressure homogenizer (AH-Basic, China) to obtain crude enzyme extracts. The crude enzyme extracts were centrifuged at 4 °C and 12,000 rpm for 30 min to remove cell debris. The supernatants containing the target proteins were filtered through a 0.22 μm membrane, loaded onto a 5 mL HisTrap HP column (GE Healthcare, USA) using an ÄKTA-purifier system, and eluted with elution buffer (20 mM sodium phosphate, 500 mM NaCl, and 500 mM imidazole, pH 7.4). An Amicon Ultra 15 mL 10K column (Merck Millipore, USA) was used for the desalting and concentration of the proteins. The purity of the proteins was verified by sodium dodecyl sulfate-polyacrylamide gel electrophoresis (SDS-PAGE). The concentrations of proteins were measured with a bradford protein assay kit (Sangon, China).

### 2.4 Assays of PheDH and LeuDH activities

The enzymatic activities of PheDH and LeuDH towards α-ketoisovalerate or α-ketoisocaproate were determined using a SpectraMax Plus384 microplate reader. The change in absorbance of NADH at 340 nm was monitored at 37 °C. The reaction mixture contained 100 mM Tris-HCl (pH 7.8), 5 mM α-ketoisovalerate or α-ketoisocaproate, 0.25 mM NADH, 200 mM NH_4_Cl, and an appropriate amount of PheDH or LeuDH.

### 2.5 l-Leucine fermentation in bioreactor

Batch fermentation was conducted in a 1-L bioreactor with a working volume of 0.8 L. Fed-batch fermentation and repeated fed-batch fermentation were performed in a 7.5-L bioreactor with a working volume of 5 L. The l-leucine fermentation was conducted at 37 °C, 500 rpm, 1.6 vvm, pH 6.8, and glucose concentration of 60 g/L. Glucose powder was supplemented when its concentration in the fermentation broth fell below 20 g/L, restoring the concentration to approximately 40–45 g/L. The pH of the fermentation broth was controlled at 6.8 by automatically feeding 25 % ammonia solution. Each cycle of repeated fed-batch fermentation was stopped before crystallization of l-leucine. A total of 4.3 L of broth was discharged from the bioreactor, and an equal volume of fresh medium was replenished using a peristaltic pump before the next cycle of fed-batch fermentation.

### 2.6 Analytical methods

The concentrations of acetoin and 2,3-butanediol were determined by gas chromatograph (GC2014C, Shimadzu, Japan) equipped with an Agilent HP-5 capillary column (0.32 mm inner diameter, 30 m length, 1.0 μm film thickness). The detector (FID) temperature was set at 280 °C, the injection volume was 1 μL, the column oven temperature was maintained at 80 °C, with an analysis time of 4 min [22]. The concentrations of organic acids like pyruvate, 2,3-dihydroxyisovalerate, 2-isopropylmalate, 3-isopropylmalate, and α-ketoisocaproate were detected using HPLC [26]. Glucose concentration was analyzed with an SBA-40D bioanalyzer. Biomass was quantified by measuring OD_600nm_. The concentrations of l-leucine and l-valine, derivatized with triethylamine and phenylisothiocyanate, were determined by HPLC (Agilent 1100, Agilent, USA) using a ZORBAX SB-C18 column (250 × 4.6 mm, Agilent, USA) as described previously [27].

## 3 Results and discussion

### 3.1 Introducing exogenous l-leucine biosynthesis pathway to achieve l-leucine accumulation

**Fig. 2:**
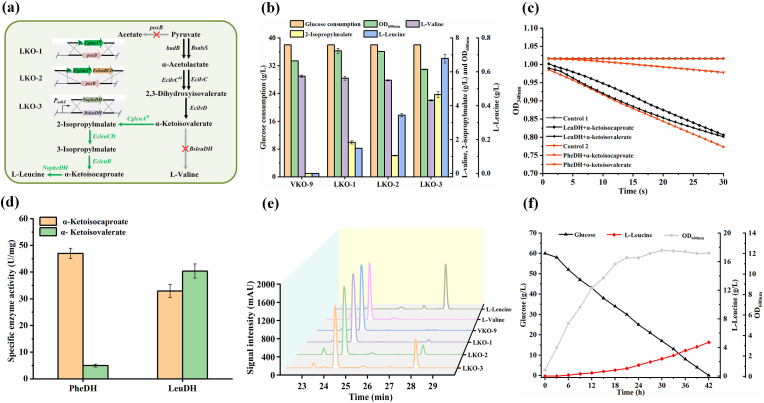
Introducing exogenous l-leucine biosynthesis pathway to accumulate l-leucine. (a) *K. oxytoca* LKO-1, LKO-2, and LKO-3 construction. (b) l-Leucine production by *K. oxytoca* VKO-9, LKO-1, LKO-2, and LKO-3 in medium with 38.0 g/L glucose. Values are the average ± SD (n = 3 independent experiments). (c) Detection of activities of PheDH and LeuDH toward different substrates. Control 1‌, LeuDH catalytic system without addition of α-ketoisocaproate or α-ketoisovalerate; Control 2‌, PheDH catalytic system without addition of α-ketoisocaproate or α-ketoisovalerate. (d) Specific activities of PheDH and LeuDH towards α-ketoisocaproate and α-ketoisovalerate. Values are the average ± SD (n = 3 independent experiments). (e) HPLC analysis of l-leucine, l-valine standards, and produced amino acids in fermented broth of strain VKO-9, LKO-1, LKO-2 and LKO-3. (f) OD_600nm_, carbon source consumption, and l-leucine production during batch fermentation of *K. oxytoca* LKO-3.

*K. oxytoca* VKO-9, an efficient producer of l-valine, was metabolic engineered for l-leucine production [23]. The isopropylmalate synthase (IPMS) catalyzes the condensation between acetyl-CoA and 2-oxoisovalerate to generate 2-isopropylmalate. Its activity determines the metabolic flux distribution between l-valine and l-leucine synthesis and is subject to feedback inhibition by l-leucine [28]. The *CgleuA*^*M*^ gene encoding l-leucine insensitive IPMS mutant (R529H and G532D) from *C. glutamicum* was inserted at the *poxB* site in *K. oxytoca* VKO-9 genome and controlled by *trc* promoter, resulting in *K. oxytoca* LKO-1 (Fig. 2a). *K. oxytoca* LKO-1 produced 0.149 g/L l-leucine but accumulated 1.84 g/L 2-isopropylmalate during shake flask fermentation (Fig. 2b). 2-Isopropylmalate can be converted into α-ketoisocaproate, the direct precursor of l-leucine, through isopropylmalate isomerase (IPMI) and isopropylmalate dehydrogenase (IPMDH) [29]. Thus, *EcleuCD* and *EcleuB* genes encoding IPMI and IPMDH from *E. coli* W3110 and *CgleuA*^*M*^ gene were assembled into an artificial operon with *trc* promoter, and then integrated into the *poxB* locus of VKO-9, generating *K. oxytoca* LKO-2 (Fig. 2a). The concentration of 2-isopropylmalate produced by *K. oxytoca* LKO-2 decreased to 1.06 g/L, while the production of l-leucine increased to 0.340 g/L (Fig. 2b).

*K. oxytoca* LKO-2 produced 5.50 g/L l-valine, the major by-product accumulated by the strain. The *BsleuDH* encoding leucine dehydrogenase (LeuDH) from *B*. *subtilis* 168 was previously inserted in *K. oxytoca* VKO-9 genome to catalyze the reductive amination of α-ketoisovalerate to generate l-valine. LeuDH was overexprssed in *E. coli* BL21 (DE3) and then purified (Fig. S1). As shown in Fig. 2c and d, the purifed LeuDH also catalyzes the generation of l-leucine from α-ketoisocaproate, but its specific activity toward α-ketoisocaproate is lower than that toward α-ketoisovalerate. Phenylalanine dehydrogenase (PheDH) from *Nocardia* sp. 239 was reported to exhibit high enzymatic activity toward α-ketoisocaproate. Importantly, its specific activity toward α-ketoisocaproate is much higher than that toward α-ketoisovalerate (Fig. 2c and d) [30]. The *NopheDH* encoding PheDH was inserted into *K. oxytoca* LKO-2 genome to replace *BsleuDH* (Fig. 2a). The obtained strain *K. oxytoca* LKO-3 produced 0.679 g/L l-leucine and 4.32 g/L l-valine (Fig. 2b). Chromatographic analysis also revealed that the introduction of l-leucine synthesis pathway can increase l-leucine production and decrease l-valine generation (Fig. 2e). Batch cultivation of *K. oxytoca* LKO-3 in a 1-L bioreactor resulted in 4.73 g/L l-leucine production from 60.0 g/L glucose (Fig. 2f).

### 3.2 Enhancing the conversion of α-ketoisovalerate into α-ketoisocaproate for improvement of l-leucine generation

**Fig. 3:**
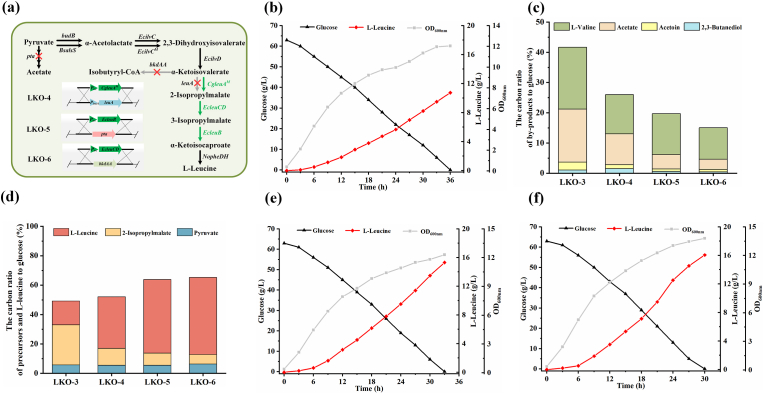
Enhancing the expression of IPMS^M^, IPMDH, and IPMI to increase l-leucine generation. (a) *K. oxytoca* LKO-4, LKO-5, and LKO-6 construction. (b) OD_600nm_, carbon source consumption, and l-leucine production during batch fermentation of *K. oxytoca* LKO-4. Values are the average ± SD (n = 3 independent experiments). (c) Carbon ratio of by-products to glucose consumption of *K. oxytoca* LKO-3, LKO-4, LKO-5, and LKO-6. (d) Carbon ratio of l-leucine or its precursors to glucose consumption of *K. oxytoca* LKO-3, LKO-4, LKO-5, and LKO-6. Values are the average of independent experiments. (e) OD_600nm_, carbon source consumption, and l-leucine production during batch fermentation of *K. oxytoca* LKO-5. (f) OD_600nm_, carbon source consumption, and l-leucine production during batch fermentation of *K. oxytoca* LKO-6. The experiments were conducted in triplicate. Two representative time-courses of *K. oxytoca* LKO-5 (e) and *K. oxytoca* LKO-6 (f) are reported herein.

The *leuABCD* operon for l-leucine production is negatively regulated by l-leucine-mediated transcription attenuation [16]. Thus, the promoter and transcription attenuation region of *leuABCD* operon in *K. oxytoca* LKO-3 were replaced with *trc* promoter. The endogenous *leuA* of *K. oxytoca* LKO-3 was also replaced with the second copy of *CgleuA*^*M*^, generating *K. oxytoca* LKO-4 (Fig. 3a). Batch fermentation of *K. oxytoca* LKO-4 was then conducted in a 1-L bioreactor. *K. oxytoca* LKO-4 produced 10.7 g/L l-leucine from 63.0 g/L glucose (Fig. 3b). Distribution of metabolic flux in *K. oxytoca* LKO-3 and *K. oxytoca* LKO-4 was also illustrated by comparison of the extracellular‌ product distribution. The carbon ratio directing to by-products decreased from 41.6 % to 26.0 %, and the carbon ratio directing to l-leucine or its precursors (2-isopropylmalate and pyruvate) increased from 49.3 % to 52.1 % (Fig. 3c and d). Theoretically, quantitative analysis of flux changes at key intracellular metabolic nodes is more intuitive for characterizing the effects of genetic engineering and thus worth attempting.

To enhance the conversion of 2-isopropylmalate to α-ketoisocaproate, the second copy of *EcleuB* and *EcleuCD* were successively integrated into the genome of *K. oxytoca* LKO-4, resulting in LKO-5 and LKO-6, respectively. As shown in Fig. 3e, 15.3 g/L of l-leucine was produced by *K. oxytoca* LKO-5 from 63.0 g/L glucose within 33 h. As shown in Fig. 3f, 16.0 g/L l-leucine was produced by *K. oxytoca* LKO-6 from 63.0 g/L glucose within 30 h. Compared to *K. oxytoca* LKO-4, the carbon ratio directing to 2-isopropylmalate in *K. oxytoca* LKO-6 decreased from 11.5 % to 6.4 %, and the carbon ratio directing to l-leucine increased to 52.4 % (Fig. 3d).

### 3.3 Modifying l-leucine transport system and increasing CgleuA^M^ expression to enhance l-leucine synthesis

**Fig. 4:**
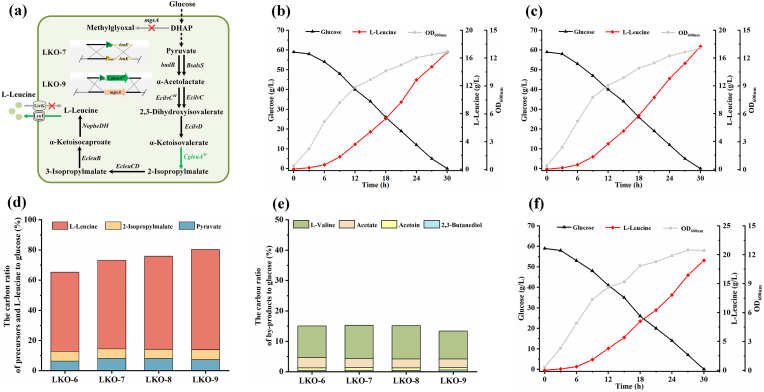
Modifying l-leucine transport and increasing copy number of *CgleuA*^*M*^ to enhance l-leucine synthesis. (a) *K. oxytoca* LKO-7, LKO-8, and LKO-9 construction. (b) OD_600nm_, carbon source consumption, and l-leucine production during batch fermentation of *K. oxytoca* LKO-7. (c) OD_600nm_, carbon source consumption, and l-leucine production during batch fermentation of *K. oxytoca* LKO-8. (d) Carbon ratio of l-leucine or its precursors to glucose consumption of *K. oxytoca* LKO-6, LKO-7, LKO-8, and LKO-9. (e) Carbon ratio of by-products to glucose consumption of *K. oxytoca* LKO-6, LKO-7, LKO-8, and LKO-9. Values are the average of independent experiments. (f) OD_600nm_, carbon source consumption, and l-leucine production during batch fermentation of *K. oxytoca* LKO-9. The experiments were conducted in triplicate. Three representative time-courses of *K. oxytoca* LKO-7 (b), *K. oxytoca* LKO-8 (c), and *K. oxytoca* LKO-9 (f) are reported herein.

Transmembrane export of l-leucine can alleviate feedback inhibition and cytotoxicity caused by high intracellular l-leucine accumulation [12,31]. To improve the l-leucine efflux in *K. oxytoca* LKO-6, the promoter of *leuE* (encoding l-leucine exporter LeuE) was replaced with the *trc* promoter, yielding *K. oxytoca* LKO-7 (Fig. 4a). To prevent l-leucine reabsorption, the *livK* (encoding l-leucine importer LivK) in *K. oxytoca* LKO-7 was deleted, resulting in *K. oxytoca* LKO-8.

*K. oxytoca* LKO-7 produced 16.8 g/L l-leucine from 59.0 g/L glucose, with a yield of 0.285 g/g (Fig. 4b). After further knockout of *livK*, the l-leucine production and yield of *K. oxytoca* LKO-8 increased to 17.7 g/L and 0.300 g/g, respectively (Fig. 4c). The carbon ratio directing to l-leucine in *K. oxytoca* LKO-8 increased from 52.4 % to 61.6 % (Fig. 4d).

As shown in Fig. 4e, 11.0 % of the carbon ratio was still directed to l-valine in *K. oxytoca* LKO-8. α-Ketoisovalerate, the substrate of IPMS, is also the direct precursor of l-valine. The accumulation of l-valine indicated that the expression of *CgleuA*^*M*^ (encoding IPMS^M^) in *K. oxytoca* LKO-8 was still insufficient to fully redirect the metabolic flux at the α-ketoisovalerate node towards l-leucine production. Thus, the third copy of *CgleuA*^*M*^ was inserted into *K. oxytoca* LKO-8 genome at *mgsA* site to enhance IPMS^M^ expression (Fig. 4a). The resulting strain *K. oxytoca* LKO-9 produced 19.0 g/L l-leucine within 30 h, with a yield of 0.322 g/g (Fig. 4f). Introducing the third copy of *CgleuA*^*M*^ in *K. oxytoca* LKO-9 further raised the carbon ratio directing to l-leucine, and the carbon ratio directing to l-valine decreased to 9.18 % (Fig. 4d and e).

### 3.4 Strengthening pyruvate condensation and 2,3-dihydroxyisovalerate dehydration to promote l-leucine production

**Fig. 5:**
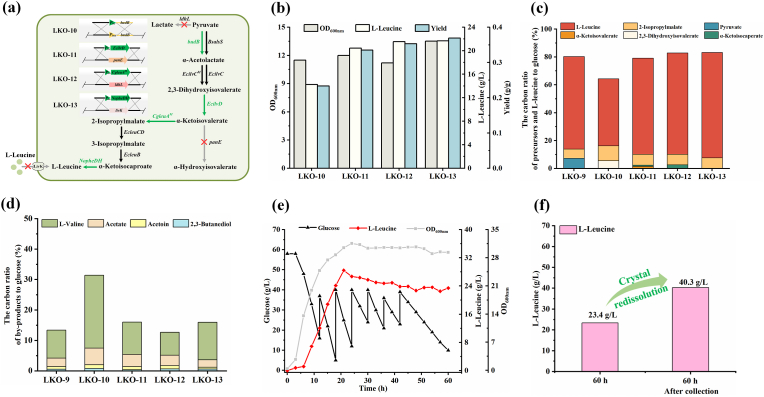
Enhancing the conversion of precursors to improve l-leucine production. (a) *K. oxytoca* LKO-10, LKO-11, LKO-12, and LKO-13 construction. (b) OD_600nm_, concentration and yield of l-leucine of *K. oxytoca* LKO-10, LKO-11, LKO-12, and LKO-13. (c) Carbon ratio of l-leucine and its precursors to glucose consumption of *K. oxytoca* LKO-9, LKO-10, LKO-11, LKO-12, and LKO-13. (d) Carbon ratio of by-products to glucose consumption of *K. oxytoca* LKO-9, LKO-10, LKO-11, LKO-12, and LKO-13. Values are the average of independent experiments. (e) OD_600nm_, carbon source consumption, and l-leucine production during fed-batch fermentation of *K. oxytoca* LKO-13. The experiments were conducted in triplicate. One representative time-course of *K. oxytoca* LKO-13 is reported herein. (f) Quantification of l-leucine before and after re-dissolution of crystal in broth after fed-batch fermentation of strain LKO-13.

Pyruvate was another l-leucine precursor accumulated by *K. oxytoca* LKO-9 (Fig. 4d). The α-acetolactate synthase (BudB) catalyzes the condensation of pyruvate, the initial reaction in l-leucine biosynthesis. The expression of *budB* in *K. oxytoca* is activated by acetate activation [32]. However, the *pta* gene responsible for acetate production was replaced by *EcleuB* in *K. oxytoca* LKO-9, which may result in insufficient BudB expression and pyruvate accumulation. Shake flask fermentation of *K. oxytoca* LKO-9 was conducted in media with or without 2 g/L acetate addition. Acetate addition obviously decreased pyruvate accumulation (from 6.01 g/L to 0.945 g/L), and increased l-leucine production (from 3.11 g/L to 4.38 g/L) of *K. oxytoca* LKO-9 (Fig. S2). Thus, the promoter of *budB* was replaced by *trc* promoter to get rid of its dependence on acetate, resulting in *K. oxytoca* LKO-10 (Fig. 5a).

Batch fermentation of *K. oxytoca* LKO-10 produced 14.2 g/L l-leucine, with a yield of 0.233 g/g (Fig. 5b). As expected, *K. oxytoca* LKO-10 no longer accumulated pyruvate (Fig. 5c). However, 2,3-dihydroxyisovalerate accumulation in the fermentation broth was observed (Fig. 5c). 2,3-Dihydroxyisovalerate can be dehydrated to α-ketoisovalerate by dihydroxyacid dehydratase (DHAD) [23]. The gene *EcilvD* encoding EcDHAD from *E. coli* W3110 was previously inserted at *ldhD* site of strain VKO-9. The α-hydroxyacid dehydrogenase encoded by *panE* in *K. pneumoniae* has been reported to reduce α-ketoisovalerate to α-hydroxyisovalerate [33]. To decrease the accumulation of 2,3-dihydroxyisovalerate and prevent the reduction of α-ketoisovalerate to α-hydroxyisovalerate, the second copy of *EcilvD* was integrated into the *panE* locus in *K. oxytoca* LKO-10 genome and controlled by *trc* promoter (Fig. 5a). The obtained strain *K. oxytoca* LKO-11 produced 20.4 g/L l-leucine, with a yield of 0.334 g/g (Fig. 5b). Neither pyruvate nor 2,3-dihydroxyisovalerate accumulation was detected in fermentation broth.

### 3.5 Increasing the expression of isopropylmalate synthase and phenylalanine dehydrogenase to facilitate l-leucine biosynthesis

Slight accumulation of α-ketoisovalerate and α-ketoisocaproate was observed during batch fermentation of *K. oxytoca* LKO-11 (Fig. 5c). To reduce the accumulation of α-ketoisovalerate, the fourth copy of *CgleuA*^*M*^ was integrated into the *ldhL* locus of *K. oxytoca* LKO-11 (Fig. 5a), generating *K. oxytoca* LKO-12. The concentration and yield of l-leucine produced by *K. oxytoca* LKO-12 increased to 21.5 g/L and 0.352 g/g, respectively (Fig. 5b). α-Ketoisovalerate accumulation was eliminated in *K. oxytoca* LKO-12, while the carbon ratio of α-ketoisocaproate increased from 0.82 % to 2.66 % (Fig. 5c).

α-Ketoisocaproate can be transformed into l-leucine via reductive amination by PheDH [30]. Thus, the second copy of *NopheDH* was integrated at *livK* site of strain LKO-12, resulting in *K. oxytoca* LKO-13 (Fig. 5a). As shown in Fig. 5b, the l-leucine production of *K. oxytoca* LKO-13 in a 1-L bioreactor reached 21.7 g/L, and the yield of l-leucine increased to 0.368 g/g.

### 3.6 Introduction of oxygen-tolerant dihydroxyacid dehydratase to enhance l-leucine production

Fed-batch fermentation of *K. oxytoca* LKO-13 was carried out in a 7.5-L bioreactor. *K. oxytoca* LKO-13 consumed 179 g/L glucose in 60 h (Fig. 5e). l-Leucine concentration reached 28.4 g/L at 21 h and it began to crystallize after 24 h. After collecting and redissolving the crystal in fermentation broth, the actual l-leucine production by *K. oxytoca* LKO-13 was determined to be 40.3 g/L (Fig. 5f). The yield of l-leucine in 7.5-L bioreactor was 0.225 g/g, which was significantly lower than that in the 1-L bioreactor (0.368 g/g).

**Fig. 6:**
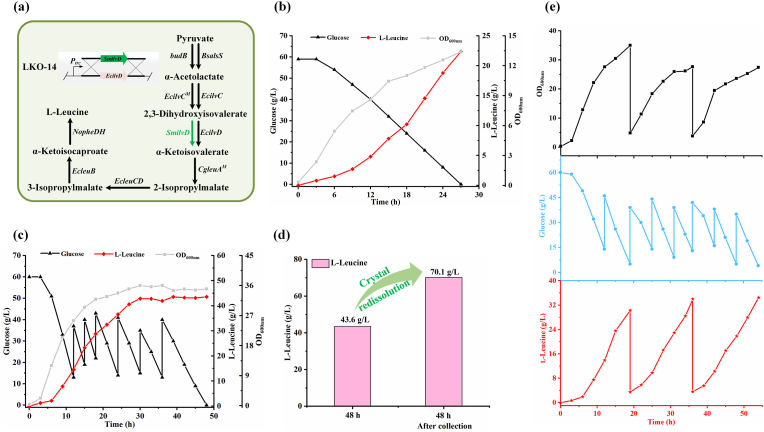
Batch fermentation, fed-batch fermentation, and repeated fed-batch fermentation of *K. oxytoca* LKO-14. (a) *K. oxytoca* LKO-14 construction strategy. (b) OD_600nm_, carbon source consumption, and l-leucine production of *K. oxytoca* LKO-14 during batch fermentation. The experiments were conducted in triplicate. One representative time-course of *K. oxytoca* LKO-14 is reported herein. (c) OD_600nm_, carbon source consumption, and l-leucine production of *K. oxytoca* LKO-14 during fed-batch fermentation. The experiments were conducted in triplicate. One representative time-course of *K. oxytoca* LKO-14 is reported herein. (d) Quantification of l-leucine before and after re-dissolution of crystal in broth after fed-batch fermentation of strain LKO-14. (e) OD_600nm_, carbon source consumption, and l-leucine production of *K. oxytoca* LKO-14 during repeated fed-batch fermentation.

The decrease in l-leucine yield in the 7.5-L bioreactor for *K. oxytoca* LKO-13 may be caused by the generation of 2,3-dihydroxyisovalerate, which began to accumulate at 24 h (Fig. S3). The genome of *K. oxytoca* LKO-13 harbored two copies of *EcilvD* encoding EcDHAD, which were controlled by *ldhD* and *trc* promoters, respectively. EcDHAD exhibits a high specific activity of 63 U/mg but its active site contains a [4Fe–4S] cluster, which is susceptible to inactivation under conditions of high oxidative stress [34]. The accumulation of 2,3-dihydroxyisovalerate may result from oxygen-induced inactivation of EcDHAD. The dihydroxy acid dehydratase from *Streptococcus mutans* (SmDHAD) exhibits a specific activity of 7.9 U/mg but its active site contains a [2Fe–2S] cluster and thus is more oxygen-tolerant [35]. As demonstrated by Flint et al., SmDHAD is stable (maintaining >80 % of its initial activity) after 72 h of incubation in air, while EcDHAD rapidly loses its activity in 24 h under identical conditions [35]. Except for preventing deactivation of EcDHAD through process optimization like precise oxygen control, directly introducing oxygen-tolerant SmDHAD may also decrease the accumulation of 2,3-dihydroxyisovalerate and increase l-leucine production. Therefore, the *EcilvD* controlled by the *trc* promoter in *K. oxytoca* LKO-13 was replaced with *SmilvD* encoding SmDHAD, resulting in *K. oxytoca* LKO-14 (Fig. 6a). Batch fermentation of *K. oxytoca* LKO-14 in 1-L bioreactor resulted in 22.4 g/L l-leucine production, with a yield of 0.379 g/g and a productivity of 0.827 g/L/h (Fig. 6b).

### 3.7 Fed-batch fermentation and repeated fed-batch fermentation for l-leucine production

Fed-batch fermentation of *K. oxytoca* LKO-14 was then carried out in 7.5-L bioreactor. It consumed 202 g/L glucose and produced 70.1 g/L l-leucine within 48 h (Fig. 6c), with a yield of 0.347 g/g and a productivity of 1.46 g/L/h (Fig. 6d). The concentrations of byproducts including 2-isopropylmalate, acetate, acetoin, and 2,3-butanediol were 4.45 g/L, 2.40 g/L, 1.52 g/L, and 1.68 g/L, respectively (Fig. S4). Importantly, the introduce of SmDHAD prevented the accumulation of 2,3-dihydroxyisovalerate, while the final concentration of l-valine was 0.887 g/L (Fig. S4). To exclude the possible damage of crystallized l-leucine toward fermentation equipment, three cycles of repeated fed-batch fermentation of strain LKO-14 was also conducted (Fig. 6e). The average concentration, yield, and productivity of l-leucine were 30.6 g/L, 0.353 g/g, and 1.70 g/L/h, respectively. A total of 30 serial subcultures of strain LKO-14 were performed in shake flask with a gap of every 12 h, and shake flask fermentation was conducted at every 10th subculture. No significant change in glucose consumption and l-leucine production in shake flask fermentation was detected (Fig. S5), indicating the stability of the production performance of *K. oxytoca* LKO-14.

Both *E. coli* and *C. glutamicum* have been metabolic engineered for l-leucine production (Table 1). Ding et al. achieved high production of l-leucine by multistep metabolic engineering of *E*. *coli*. The obtained strain *E*. *coli* LXH-21 produced 63.29 g/L l-leucine, which is the highest l-leucine production among previous reports [12]. Recently, Hao et al. constructed a plasmid-free l-leucine producing strain *E*. *coli* LEU27. *E*. *coli* LEU27 produced 55 g/L of l-leucine in 48 h, with a yield of 0.23 g/g and a productivity of 1.15 g/L/h [13]. In this study, *K. oxytoca* LKO-14 was constructed through redirecting the metabolic flux in *K. oxytoca* VKO-9, from l-valine production to l-leucine synthesis. *K. oxytoca* LKO-14 produced 70.1 g/L l-leucine within 48 h, with a yield of 0.347 g/g and a productivity of 1.46 g/L/h. The key genes for l-leucine generation were inserted into *K. oxytoca* LKO-14 genome and constitutively expressed, making it a promising candidate for low cost and stable l-leucine production. Importantly, the final concentration of l-valine of *K. oxytoca* LKO-14 was only 0.887 g/L, which may reduce the separation cost and be beneficial for downstream process of industrial l-leucine production.

**Table 1:** Comparison of l-leucine production by different microorganisms.

Strain	Titer (g/L)	Yield (g/g)	Productivity (g/L/h)	Plasmid	Inducer	Reference
*E. coli* LXH-21	63.29	0.37	2.64	pTrc99aΔ*lacI-leuA*^*CP*^*BCD*	NR	[12]
*E. coli* LEU-27	55	0.23	1.15	NR^b^	NR	[13]
*C. glutamicum* MV-LeuF2	24	0.19	0.428	NR	NR	[29]
*C. glutamicum* JL-51	40.11	0.25	0.59	pECXK99E-*acscobB*	IPTG	[36]
*C. glutamicum* Leu-9	54.3	NM^a^	0.754	pECXK99E-*leuAilvBNCE*	IPTG	[17]
*K. oxytoca* LKO-14	70.1	0.347	1.46	NR	NR	This study

a Not mentioned.

b Not required.

## 4 Conclusions

In summary, the metabolic flux in *K. oxytoca* VKO-9, an l-valine producing strain, was redirected to l-leucine synthesis. The obtained strain *K. oxytoca* LKO-14 efficiently produced l-leucine from glucose via fed-batch fermentation, with a concentration, yield and productivity of 70.1 g/L, 0.347 g/g and 1.46 g/L/h, respectively. The average concentration, yield, and productivity of l-leucine in repeated fed-batch fermentation were 30.6 g/L, 0.353 g/g, and 1.70 g/L/h, respectively. The plasmid free, inducer independent, and low l-valine generation characteristics of *K. oxytoca* LKO-14 made it a promising alternative for industrial l-leucine production.

## CRediT authorship contribution statement

**Weikang Sun:** Writing – original draft, Investigation, Formal analysis, Data curation. **Qiaoyue Yang:** Writing – original draft, Investigation, Data curation. **Shuo Wang:** Investigation, Formal analysis. **Lingru Gong:** Investigation. **Zhi Zhou:** Resources, Project administration. **Mingyuan Liu:** Formal analysis. **Xiaoxu Tan:** Formal analysis. **Qianjin Kang:** Resources, Project administration. **Wensi Meng:** Project administration, Formal analysis. **Yidong Liu:** Resources, Project administration, Formal analysis. **Zhaoqi Kang:** Project administration, Data curation. **Ping Xu:** Validation, Supervision. **Cuiqing Ma:** Validation, Supervision, Resources, Project administration, Data curation, Conceptualization. **Chao Gao:** Validation, Supervision, Resources, Project administration, Formal analysis, Data curation, Conceptualization. **Chuanjuan Lü:** Validation, Supervision, Resources, Project administration, Data curation, Conceptualization.

## Declaration of competing interest

The authors declare that they have no known competing financial interests or personal relationships that could have appeared to influence the work reported in this paper.
