# Supplementary material for: Multistep metabolic engineered Klebsiella oxytoca for efficient l-leucine production
Source: Synth Syst Biotechnol. 2026 Feb 6;13:163–71. doi: 10.1016/j.synbio.2026.01.024 (PMC12907686; doi:10.1016/j.synbio.2026.01.024)
Supplement: Multimedia component 1 [file mmc1.doc]

**Multistep metabolic engineered *Klebsiella oxytoca* for efficient L-leucine production**

Weikang Sun^a, 1^, Qiaoyue Yang^a, 1^, Shuo Wang^a, 1^, Lingru Gong^a^, Zhi Zhou^b^, Mingyuan Liu^a^, Xiaoxu Tan^a^, Qianjin Kang^c^, Wensi Meng^a^, Yidong Liu^a^, Zhaoqi Kang^a^, Ping Xu^c^, Cuiqing Ma^a^, Chao Gao^a^, *, Chuanjuan Lü^a^, *

^a^State Key Laboratory of Microbial Technology, Shandong University, Qingdao, China

^b^Zibo Integrated Traditional Chinese and Western Medicine Hospital, Zibo, China

^c^State Key Laboratory of Microbial Metabolism, Shanghai Jiao Tong University, Shanghai, China

***Corresponding authors:**

Mailing address for C. Gao: State Key Laboratory of Microbial Technology, Shandong University, Qingdao 266237, China, Tel/Fax: +86-532-58631559, E-mail: jieerbu@sdu.edu.cn.

Mailing address for C. Lü: State Key Laboratory of Microbial Technology, Shandong University, Qingdao 266237, China, Tel/Fax: +86-532-58631559, E-mail: chuanjuanlv@sdu.edu.cn .

^1^ These authors contributed equally.


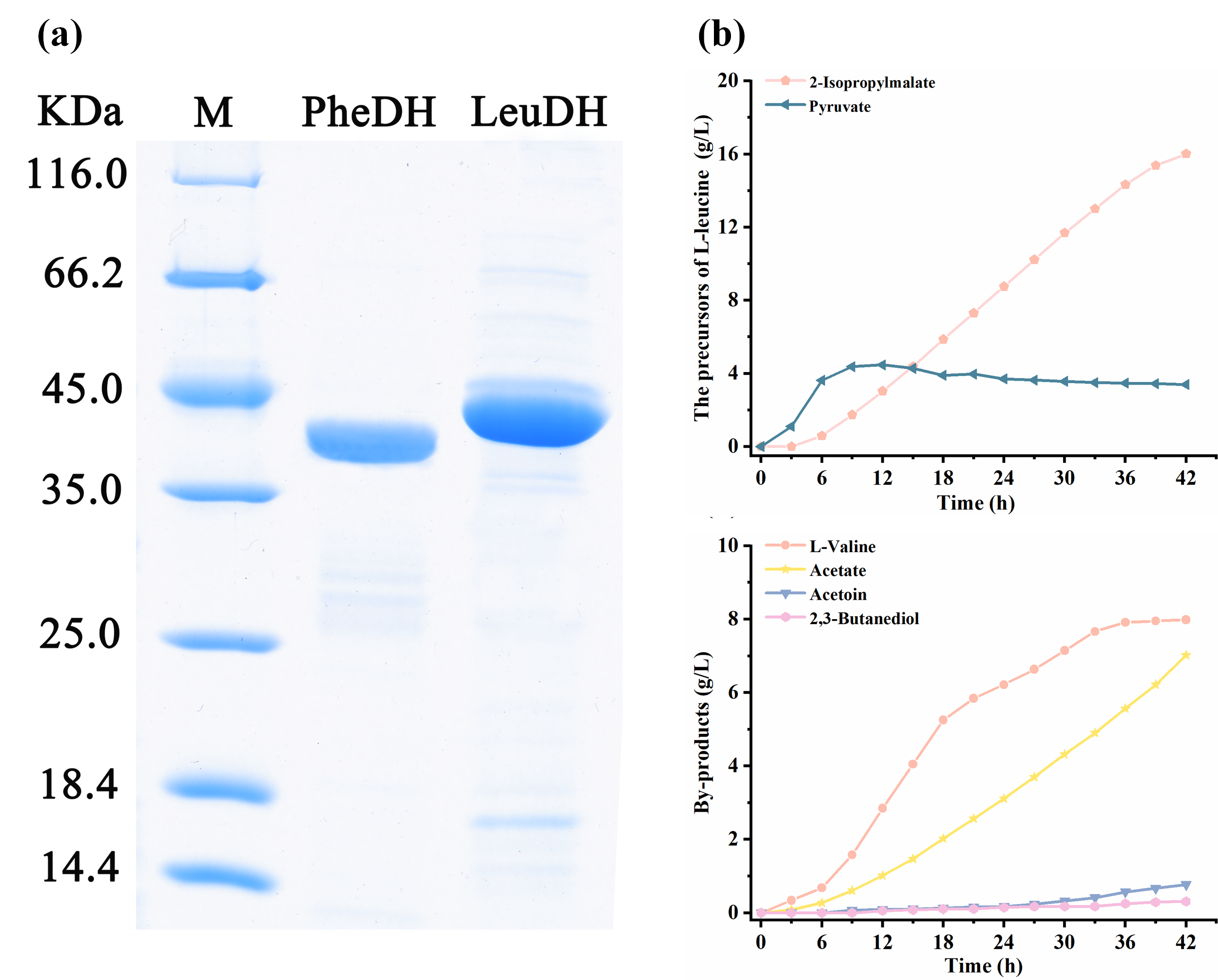


Fig. S1 SDS-PAGE analysis of the purified PheDH and LeuDH.


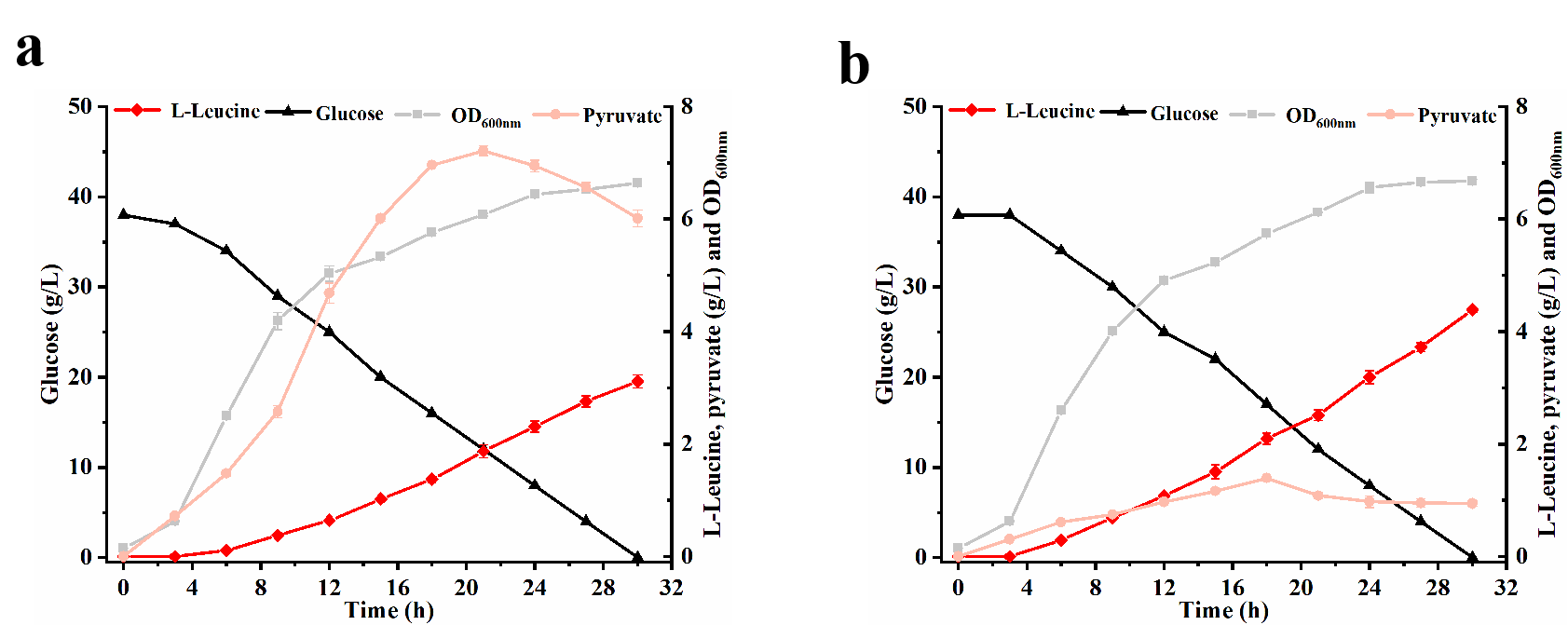


Fig. S2 (a) Biomass, glucose consumption, pyruvate and L-leucine production by *K. oxytoca* LKO-9 in fermentation medium containing 38.0 g/L glucose at 37 ℃ and 180 rpm. Data shown are mean ± s.d. (*n =* 3 independent experiments). (b) Biomass, glucose consumption, pyruvate and L-leucine production by *K. oxytoca* LKO-9 in fermentation medium containing 38.0 g/L glucose and 2 g/L acetate at 37 ℃ and 180 rpm. Data shown are mean ± s.d. (*n =* 3 independent experiments).





Fig. S3 By-products and L-leucine precursors generation of *K. oxytoca* LKO-13 during fed-batch fermentation.


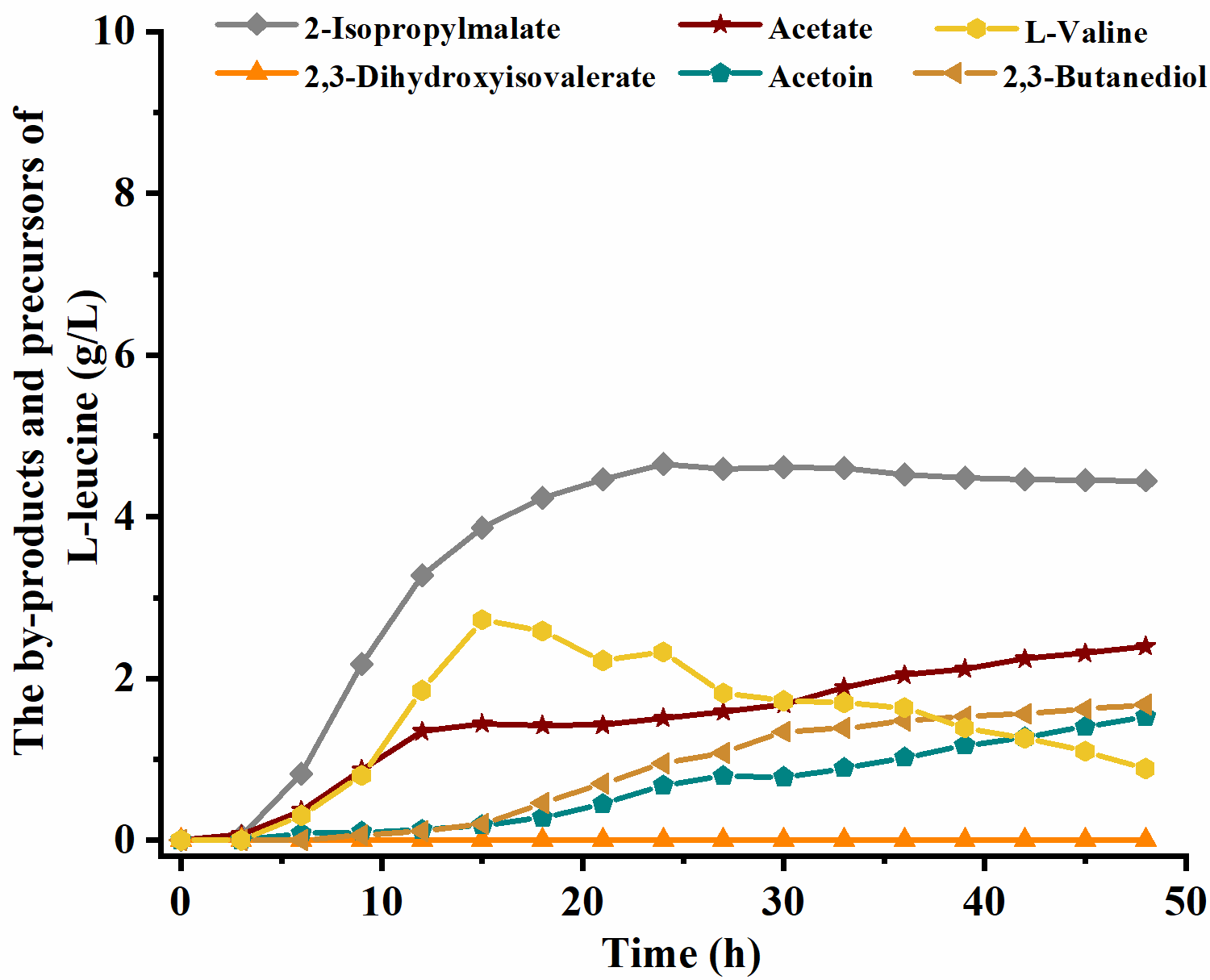


Fig. S4 By-products and L-leucine precursors generation of *K. oxytoca* LKO-14 during fed-batch fermentation.


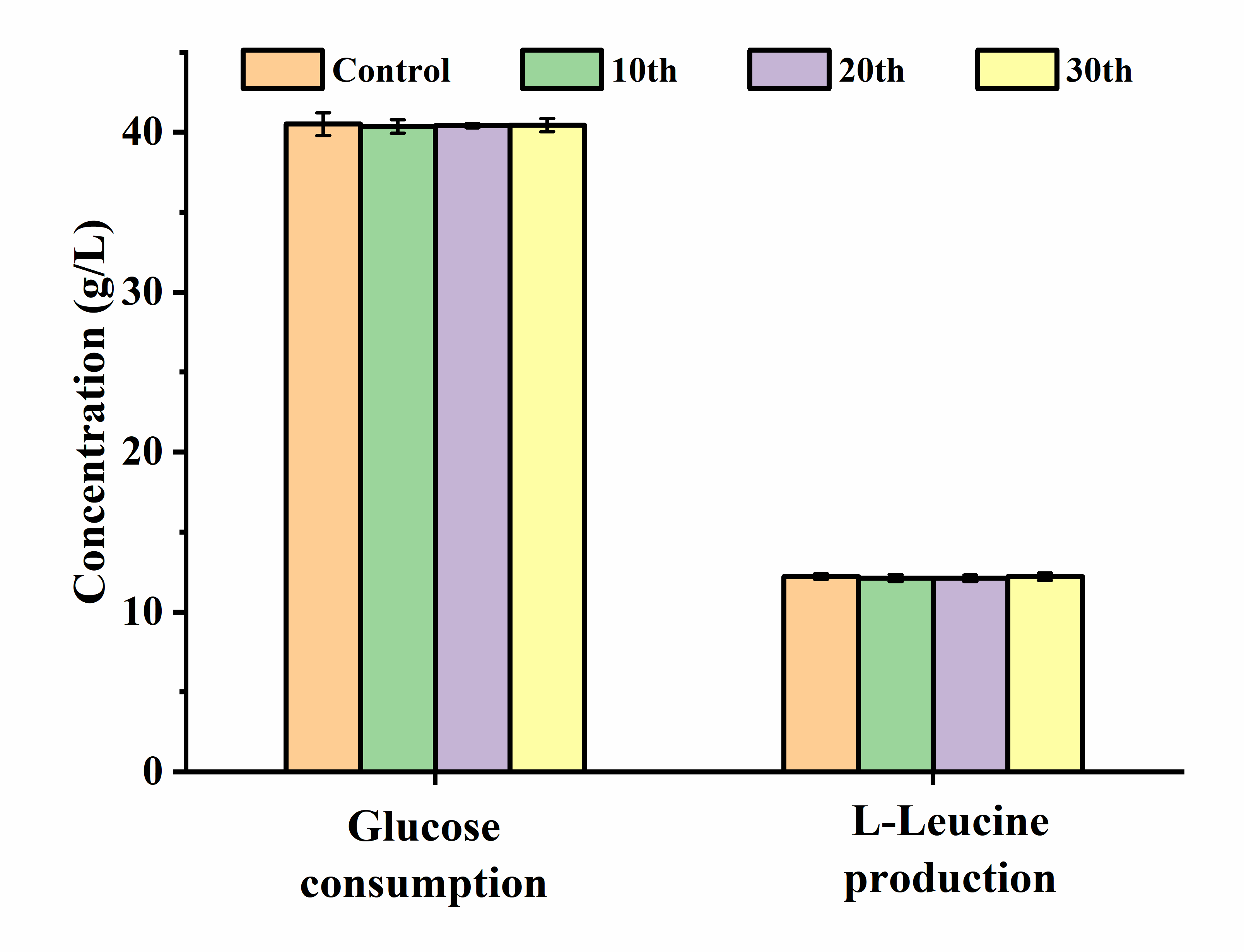


Fig. S5 Stability of the production performance of *K. oxytoca* LKO-14 during serial subcultures. Shake flask fermentation of different subcultures of *K. oxytoca* LKO-14 was conducted at 37°C and 180 rpm for 36 h. Values are the average±SD (n=3 independent experiments).

Table S1 Bacterial strains and plasmids used in this study.

| **Strain or plasmid** | **Relevant characteristics^a^** | **Origin** |
| --- | --- | --- |
| **Strain** | | |
| *C. glutamicum* ATCC13032 | Wild-type | Lab stock |
| *E. coli* W3110 | Wild-type | Lab stock |
| *E. coli* BL21(DE3) | *E. coli* B, F' *hsdSgal1* DE3, with IPTG-inducible chromosomal T7 RNA polymerase, strain for protein overexpression | Lab stock |
| *E. coli* BL21(DE3)/ pET28a-*leuDH* | *E. coli* BL21(DE3) expressing LeuDH | This study |
| *E. coli* BL21(DE3)/ pET28a-*pheDH* | *E. coli* BL21(DE3) expressing PheDH | This study |
| *Klebsiella oxytoca* PDL-0 | Wild-type | Lab stock |
| *K*. *oxytoca* VKO-9 | *K. oxytoca* PDL-0 Δ*pox*Δ*pta*Δ*frdA*Δ*ldhD*::*ilvD*Δ*pflB*::*brnFE*Δ*adhE*::*leuDH*Δ*budA*::*ilvC^M^*Δ*budC*::*alsS*Δ*gldA*::*ilvC* | Lab stock |
| *K. oxytoca* LKO-1 | *K. oxytoca* VKO-9 Δ*poxB*::P_trc_*-CgleuA^M^* | This study |
| *K. oxytoca* LKO-2 | *K. oxytoca* VKO-9 Δ*poxB*::P_trc_*-CgleuA^M^*-*EcleuBCD* | This study |
| *K. oxytoca* LKO-3 | *K. oxytoca* LKO-2 Δ*leuDH*::*pheDH* | This study |
| *K. oxytoca* LKO-4 | *K. oxytoca* LKO-3 ΔP_leu_-*leuA*::P_trc_-*CgleuA^M^* | This study |
| *K. oxytoca* LKO-5 | *K. oxytoca* LKO-4 Δ*pta*::P_trc_-*EcleuB* | This study |
| *K. oxytoca* LKO-6 | *K. oxytoca* LKO-5 Δ*bkdAA*::P_trc_-*EcleuCD* | This study |
| *K. oxytoca* LKO-7 | *K. oxytoca* LKO-6 ΔP_leuE_::P_trc_-*leuE* | This study |
| *K. oxytoca* LKO-8 | *K. oxytoca* LKO-7 Δ*livK* | This study |
| *K. oxytoca* LKO-9 | *K. oxytoca* LKO-8 Δ*mgsA*::P_trc_-*CgleuA^M^* | This study |
| *K. oxytoca* LKO-10 | *K. oxytoca* LKO-9 ΔP_bud_::P_trc_-*budB* | This study |
| *K. oxytoca* LKO-11 | *K. oxytoca* LKO-10 Δ*panE*::P_trc_-*ilvD* | This study |
| *K. oxytoca* LKO-12 | *K. oxytoca* LKO-11 Δ*ldhL*::P_trc_-*CgleuA^M^* | This study |
| *K. oxytoca* LKO-13 | *K. oxytoca* LKO-12 Δ*livK*::P_trc_-*pheDH* | This study |
| *K. oxytoca* LKO-14 | *K. oxytoca LKO-13 ΔPtrc-ilvD::Ptrc-SmilvD* | This study |
| **Plasmid** | | |
| pEcCas | Kan^r^, vector for constitutive expression of Cas9 and inducible expression of λ-Red recombinase, *sacB*, P_rhaB_-sgRNA-pMB1, pSC101 | Addgene |
| pEcCas_Cm_ | Cm^r^, vector for constitutive expression of Cas9 and inducible expression of λ-Red recombinase, *sacB*, P_rhaB_-sgRNA-pMB1, pSC101, derived from pEcCas | Lab stock |
| pEcgRNA | Spe^r^, gRNA expression vector | Addgene |
| pET28a-*leuDH* | Kan^r^, pET28a contained the *leuDH* gene from *Bacillus subtilis* 168 | Lab stock |
| pET28a-*pheDH*^b^ | Kan^r^, pET28a contained the *pheDH* gene from *Nocardia* sp*.* 239 | Lab stock |
| pET28a-*SmilvD*^b^ | Kan^r^, pET28a contained the *SmilvD* gene from *Streptococcus mutans* | Lab stock |
| pEcgRNA-Δ*poxB* | Spe^r^, pEcgRNA derivative for transcribing sgRNA to replace *poxB* with P_trc_*-CgleuA^M^* or P_trc_*-CgleuA^M^*-*EcleuBCD* | This study |
| pEcgRNA-Δ*leuDH* | Spe^r^, pEcgRNA derivative for transcribing sgRNA to replace *leuDH* with *pheDH* | This study |
| pEcgRNA-Δ*leuA* | Spe^r^, pEcgRNA derivative for transcribing sgRNA to replace P_leu_-*leuA* with P_trc_-*CgleuA^M^* | This study |
| pEcgRNA-Δ*pta* | Spe^r^, pEcgRNA derivative for transcribing sgRNA to replace *pta* with P_trc_-*EcleuB* | This study |
| pEcgRNA-Δ*bkdAA* | Spe^r^, pEcgRNA derivative for transcribing sgRNA to replace *bkdAA* with P_trc_-*EcleuCD* | This study |
| pEcgRNA-ΔP_leuE_::P_trc_ | Spe^r^, pEcgRNA derivative for transcribing sgRNA to replace the *leuE* promoter with *trc* promoter | This study |
| pEcgRNA-Δ*livK* | Spe^r^, pEcgRNA derivative for transcribing sgRNA to knock out *livK* | This study |
| pEcgRNA-Δ*mgsA* | Spe^r^, pEcgRNA derivative for transcribing sgRNA to replace the *mgsA* with P_trc_-*CgleuA^M^* | This study |
| pEcgRNA-ΔP_bud_::P_trc_ | Spe^r^, pEcgRNA derivative for transcribing sgRNA to replace the *bud* promoter with *trc* promoter | This study |
| pEcgRNA-Δ*panE* | Spe^r^, pEcgRNA derivative for transcribing sgRNA to replace the *panE* with P_trc_-*ilvD* | This study |
| pEcgRNA-Δ*ldhL* | Spe^r^, pEcgRNA derivative for transcribing sgRNA to replace the *ldhL* with P_trc_-*CgleuA^M^* | This study |
| pEcgRNA-Δ*livK* | Spe^r^, pEcgRNA derivative for transcribing sgRNA to replace the truncated *livK* with P_trc_-*pheDH* | This study |
| pEcgRNA-Δ*ilvD* | Spe^r^, pEcgRNA derivative for transcribing sgRNA to replace the P_trc_-*ilvD* with P_trc_-*SmilvD* | This study |

^a^Kan^r^, kanamycin resistant; Cm^r^, chloramphenicol resistant; Spe^r^, spectinomycin resistant.

^b^The plasmids pET28a-*pheDH* and pET28a-*SmilvD* were synthesized by General Biosystem (Anhui) Co., Lt.

Table S2 The primers used in this study.

| **Primer** | **Sequence (5′-3′)** |
| --- | --- |
| gRNA-*poxB*-1 | TAGTCTGTGAACTGGTCTCAACCC |
| gRNA-*poxB*-2 | AAACGGGTTGAGACCAGTTCACAG |
| Δ*poxB*::P_trc_-*CgleuA^M^*-1 | GGTATTTCACACCGCATATGGCTGCTGCAAAACCAAAGTG |
| Δ*poxB*::P_trc_-*CgleuA^M^*-2 | GAAATTCCACACATTATACGAGCCGGATGATTAATTGTCAAATGGTTAAATCGCTGTTA |
| Δ*poxB*::P_trc_-*CgleuA^M^*-3 | TAATGTGTGGAATTTCACACAGGAAACAGACCATGGAATTCATGTCTCCTAACGATGCA |
| Δ*poxB*::P_trc_-*CgleuA^M^*-4 | AAGCGGCCAGTGGGTCGTTGCCGTGGCCATCGACGGTGA |
| Δ*poxB*::P_trc_-*CgleuA^M^*-5 | TCACCGTCGATGGCCACGGCAACGACCCACTGGCCGCTT |
| Δ*poxB*::P_trc_-*CgleuA^M^*-6 | ATGAATTGAAAAGGATATTTTTAAACGCCGCCAGCCAGGA |
| Δ*poxB*::P_trc_-*CgleuA^M^*-7 | GCTGGCGGCGTTTAAAAATATCCTTTTCAATTCATACGCA |
| Δ*poxB*::P_trc_-*CgleuA^M^*-8 | GACTGAGCTAGCGGTTTGATTTTGGCCGCGAC |
| Δ*poxB*::P_trc_-*CgleuA^M^*-*EcleuBCD*-1 | GGTATTTCACACCGCATATGGCTGCTGCAAAACCAAAGTG |
| Δ*poxB*::P_trc_-*CgleuA^M^*-*EcleuBCD*-2 | CCGGATGATTAATTGTCAAATGGTTAAATCGCTGTTAAA |
| Δ*poxB*::P_trc_-*CgleuA^M^*-*EcleuBCD*-3 | TTTAACAGCGATTTAACCATTTGACAATTAATCATCCGG |
| Δ*poxB*::P_trc_-*CgleuA^M^*-*EcleuBCD*-4 | ATATGGTAATTCTTCGACATTAAACGCCGCCAGCCAGGAC |
| Δ*poxB*::P_trc_-*CgleuA^M^*-*EcleuBCD*-5 | GTCCTGGCTGGCGGCGTTTAATGTCGAAGAATTACCATAT |
| Δ*poxB*::P_trc_-*CgleuA^M^*-*EcleuBCD*-6 | GTATGAATTGAAAAGGATATTTTTAATTCATAAACGCAGGTTGT |
| Δ*poxB*::P_trc_-*CgleuA^M^*-*EcleuBCD*-7 | ACAACCTGCGTTTATGAATTAAAAATATCCTTTTCAATTCATAC |
| Δ*poxB*::P_trc_-*CgleuA^M^*-*EcleuBCD*-8 | GACTGAGCTAGCGGTTTGATTTTGGCCGCGAC |
| gRNA-*leuDH*-1 | TAGTATGAGACAGACTATGTCACA |
| gRNA-*leuDH*-2 | AAACTGTGACATAGTCTGTCTCAT |
| Δ*leuDH*::*pheDH*-1 | AGCTCGGTACCCGGGGATCCGGAAAATGCCCCAGCAGCACA |
| Δ*leuDH*::*pheDH*-2 | TTTCACTTCTTCGTGTTCCATAATGCTCTCCTGATAATGTTA |
| Δ*leuDH*::*pheDH*-3 | TAACATTATCAGGAGAGCATTATGGAACACGAAGAAGTGAAA |
| Δ*leuDH*::*pheDH*-4 | ACTGACTTTACGGCTGTGGAATTAGCTCAGACGGGCTGCAAC |
| Δ*leuDH*::*pheDH*-5 | GTTGCAGCCCGTCTGAGCTAATTCCACAGCCGTAAAGTCAGT |
| Δ*leuDH*::*pheDH*-6 | AGGTCGACTCTAGAGGATCCAAGCACAAAAGGGCAATAGT |
| Δ*poxB*::P_trc_-*CgleuA^M^*-6 | ATGAATTGAAAAGGATATTTTTAAACGCCGCCAGCCAGGA |
| gRNA-*leuA*-1 | TAGTCCATGACGACTTAGGCATCG |
| gRNA-*leuA*-2 | AAACCGATGCCTAAGTCGTCATGG |
| ΔP_leu_-*leuA*::P_trc_-*CgleuA^M^*-1 | TACTAGTTGAGACCGCAGGCAAACTCAGGGCGAC |
| ΔP_leu_-*leuA*::P_trc_-*CgleuA^M^*-2 | GCCGGATGATTAATTGTCAAATGTTAAAAGAAAATACTGA |
| ΔP_leu_-*leuA*::P_trc_-*CgleuA^M^*-3 | TCAGTATTTTCTTTTAACATTTGACAATTAATCATCCGGC |
| ΔP_leu_-*leuA*::P_trc_-*CgleuA^M^*-4 | ATATGGTAATTCTTCGACATGTCGACTTAAACGCCGCCAG |
| ΔP_leu_-*leuA*::P_trc_-*CgleuA^M^*-5 | CTGGCGGCGTTTAAGTCGACATGTCGAAGAATTACCATAT |
| ΔP_leu_-*leuA*::P_trc_-*CgleuA^M^*-6 | TAAAACCGAGACCCGATGCCTAAGTCGTCATGGCGCAATATGGGCGATACGCT |
| gRNA-*pta*-1 | TAGTCCACGCCAACGCTCAGGATG |
| gRNA-*pta*-2 | AAACCATCCTGAGCGTTGGCGTGG |
| Δ*pta*::P_trc_-*EcleuB*-1 | CACCGCATATGCTGGATCCCACTGGAAGGTCTGGTTAT |
| Δ*pta*::P_trc_-*EcleuB*-2 | GAAATTCCACACATTATACGAGCCGGATGATTAATTGTCAATCAGGCGGTCAGACGGT |
| Δ*pta*::P_trc_-*EcleuB*-3 | ATAATGTGTGGAATTTCACACAGGAAACAGACCATGGAATTCATGTCGAAGAATTACCA |
| Δ*pta*::P_trc_-*EcleuB*-4 | TTTTTCATGCAGCGGGCAGATTACACCCCTTCTGCTACA |
| Δ*pta*::P_trc_-*EcleuB*-5 | TGTAGCAGAAGGGGTGTAATCTGCCCGCTGCATGAAAAA |
| Δ*pta*::P_trc_-*EcleuB*-6 | CGCCGGGTTCGACGTTTACCAT |
| gRNA-*bkdAA*-1 | TAGTGTCTCTTTTACCGGTGACGG |
| gRNA-*bkdAA*-2 | AAACCCGTCACCGGTAAAAGAGAC |
| Δ*bkdAA*::P_trc_-*EcleuCD*-1 | ACACCGCATATGCTGGATCCCGGAAAGTAGCCGCTGTACA |
| Δ*bkdAA*::P_trc_-*EcleuCD*-2 | TGTGAAATTCCACACATTATACGAGCCGGATGATTAATTGTCAAATGCAGCGACGCCGTC |
| Δ*bkdAA*::P_trc_-*EcleuCD*-3 | ATAATGTGTGGAATTTCACACAGGAAACAGACCATGGAATTCATGGCTAAGACGTTATA |
| Δ*bkdAA*::P_trc_-*EcleuCD*-4 | ATAGGCATTGTTATCACCCTTAATTCATAAACGCAGGT |
| Δ*bkdAA*::P_trc_-*EcleuCD*-5 | AACCTGCGTTTATGAATTAAGGGTGATAACAATGCCTAT |
| Δ*bkdAA*::P_trc_-*EcleuCD*-6 | ATACCTAGGACTGAGCTAGCCATAAGGCGTCGATGGCAC |
| gRNA-P_leuE_-1 | TAGTATATTTTTTGTTGTAAGCGA |
| gRNA-P_leuE_-2 | AAACTCGCTTACAACAAAAAATAT |
| ΔP_leuE_::P_trc_-*leuE*-1 | ACACCGCATATGCTGGATCCAGGCGTGATGGTCGTTGGCG |
| ΔP_leuE_::P_trc_-*leuE*-2 | GTGAAATTCCACACATTATACGAGCCGGATGATTAATTGTCAATTAATAGAGCGAAGTT |
| ΔP_leuE_::P_trc_-*leuE*-3 | ATAATGTGTGGAATTTCACACAGGAAACAGACCATGGAATTCGTGTTCGCTGAGTTTGG |
| ΔP_leuE_::P_trc_-*leuE*-4 | CCCAACGATTACGACTGGAGCGTCGCC |
| gRNA-*livK*-1 | TAGTCCGGTCTGGACTCTTCGCAG |
| gRNA-*livK*-2 | AAACCTGCGAAGAGTCCAGACCGG |
| Δ*livK*-1 | ATGGTAAAAGTAGCGTTGTT |
| Δ*livK*-2 | GTTTTCAGGCCCACGGAGCGAGAGCAGAGGTGGCCGAT |
| Δ*livK*-3 | ATCGGCCACCTCTGCTCTCGCTCCGTGGGCCTGAAAAC |
| Δ*livK*-4 | TTATTTCGCGAGGGTGGATG |
| gRNA-*mgsA*-1 | TAGTGAGGTGCATGCGATGCTGAG |
| gRNA-*mgsA*-2 | AAACCTCAGCATCGCATGCACCTC |
| Δ*mgsA*::P_trc_-*CgleuA^M^*-1 | ACACCGCATATGCTGGATCCTTAACGGGCTAATCCTAATC |
| Δ*mgsA*::P_trc_-*CgleuA^M^*-2 | GCCGGATGATTAATTGTCAATTTGGCCTGTAGACAGATTA |
| Δ*mgsA*::P_trc_-*CgleuA^M^*-3 | TAATCTGTCTACAGGCCAAATTGACAATTAATCATCCGGC |
| Δ*mgsA*::P_trc_-*CgleuA^M^*-4 | GTGGCCGCCAGCGGGGCTTAAACGCCGCCAGCCAGGACTGCCTCG |
| Δ*mgsA*::P_trc_-*CgleuA^M^*-5 | TGGCTGGCGGCGTTTAAGCCCCGCTGGCGGCCACG |
| Δ*mgsA*::P_trc_-*CgleuA^M^*-6 | ATACCTAGGACTGAGCTAGCAACAGCCTGGTCGCAGGGGA |
| gRNA-P_bud_-1 | TAGTTTTCTGATATTCGTTGAACG |
| gRNA-P_bud_-2 | AAACCGTTCAACGAATATCAGAAA |
| ΔP_bud_::P_trc_-*budB*-1 | ACACCGCATATGCTGGATCCTCAGCTCGCTATGGGACGAC |
| ΔP_bud_::P_trc_-*budB*-2 | GAAATTCCACACATTATACGAGCCGGATGATTAATTGTCAAATGGAACTTCGTTATCTG |
| ΔP_bud_::P_trc_-*budB*-3 | ATAATGTGTGGAATTTCACACAGGAAACAGACCATGGAATTCATGGCTAACTACTTCAA |
| ΔP_bud_::P_trc_-*budB*-4 | TATACCTAGGACTGAGCTAGCGCCCAGGCTTTGGCAATCG |
| gRNA-*panE*-1 | TAGTCCCGGCGAAAGCCTATCCTG |
| gRNA-*panE*-2 | AAACCAGGATAGGCTTTCGCCGGG |
| Δ*panE*::P_trc_-*ilvD*-1 | CACCGCATATGCTGGATCCCACGAACTCGTCCGGCACT |
| Δ*panE*::P_trc_-*ilvD*-2 | TGAAATTCCACACATTATACGAGCCGGATGATTAATTGTCAAATTGACGCTACAGAATGG |
| Δ*panE*::P_trc_-*ilvD*-3 | ATAATGTGTGGAATTTCACACAGGAAACAGACCATGGAATTCATGCCTAAGTACCGTTCC |
| Δ*panE*::P_trc_-*ilvD*-4 | ATCAGTGCCGACGCGCTCATTTAACCCCCCAGTTTCGATT |
| Δ*panE*::P_trc_-*ilvD*-5 | AATCGAAACTGGGGGGTTAAATGAGCGCGTCGGCACTGAT |
| Δ*panE*::P_trc_-*ilvD*-6 | ACCTAGGACTGAGCTAGCCACCAGCTGTGAAGCGAC |
| gRNA-*ldhL*-1 | TAGTTTCGGAAGACGCCCGCGATG |
| gRNA-*ldhL*-2 | AAACCATCGCGGGCGTCTTCCGAA |
| Δ*ldhL*::P_trc_-*CgleuA^M^*-1 | CACCGCATATGCTGGATCCAACCGCTGAAGACGCTGCTC |
| Δ*ldhL*::P_trc_-*CgleuA^M^*-2 | GCCGGATGATTAATTGTCAATCACAAGTTCTCCCTGGAAT |
| Δ*ldhL*::P_trc_-*CgleuA^M^*-3 | CCAGGGAGAACTTGTGATTGACAATTAATCATCCGGCTCG |
| Δ*ldhL*::P_trc_-*CgleuA^M^*-4 | GATGGCCTGATGAGGACGTTAAACGCCGCCAGCCAGGA |
| Δ*ldhL*::P_trc_-*CgleuA^M^*-5 | CTGGCTGGCGGCGTTTAACGTCCTCATCAGGCCATCCG |
| Δ*ldhL*::P_trc_-*CgleuA^M^*-6 | ATACCTAGGACTGAGCTAGCATCATCGGAATACGGATTTT |
| gRNA-*livK*-3 | TAGTGCAGGCGTCATCGTACTCCA |
| gRNA-*livK*-4 | AAACTGGAGTACGATGACGCCTGC |
| Δ*livK*::P_trc_-*pheDH*-1 | ACCGCATATGCTGGATCCTGCATAAACGCCGCCATCAC |
| Δ*livK*::P_trc_-*pheDH*-2 | TGAAATTCCACACATTATACGAGCCGGATGATTAATTGTCAATATCTTTAATGGTTTAGC |
| Δ*livK*::P_trc_-*pheDH*-3 | ATAATGTGTGGAATTTCACACAGGAAACAGACCATGGAATTCATGGAACTTTTTAAATAT |
| Δ*livK*::P_trc_-*pheDH*-4 | GGATAACTTGTCTTATGGCTTAACGTCTGCTTAATACACT |
| Δ*livK*::P_trc_-*pheDH*-5 | AGTGTATTAAGCAGACGTTAAGCCATAAGACAAGTTATCCC |
| Δ*livK*::P_trc_-*pheDH*-6 | ATACCTAGGACTGAGCTAGCCGCGCCGATCACAAACGTCA |
| gRNA-*ilvD*-1 | TAGTTACCGATAACACCACCAGCA |
| gRNA-*ilvD*-2 | AAACTGCTGGTGGTGTTATCGGTA |
| ΔP_trc_-*ilvD*::P_trc_-*SmilvD*-1 | TATGACATGATTACGAATTCCAGCAGATTGGCGTCTCCGA |
| ΔP_trc_-*ilvD*::P_trc_-*SmilvD*-2 | AGGGTCTTTTTATCGGTCATGAATTCCATGGTCTGTTTCC |
| ΔP_trc_-*ilvD*::P_trc_-*SmilvD*-3 | GGAAACAGACCATGGAATTCATGACCGATAAAAAGACCCT |
| ΔP_trc_-*ilvD*::P_trc_-*SmilvD*-4 | CGGATGGCCTGATGAGGACGTTATTTTTTGCCGGTTTCTT |
| ΔP_trc_-*ilvD*::P_trc_-*SmilvD*-5 | AAACCGGCAAAAAATAACGTCCTCATCAGGCCATCC |
| ΔP_trc_-*ilvD*::P_trc_-*SmilvD*-6 | AGGTCGACTCTAGAGGATCCTTCTGCAATCCGTGACCCTT |
| pET28a-*leuDH*-check1 | ATGGAACTTTTTAAATATAT |
| pET28a-*leuDH*-check2 | TTAACGTCTGCTTAATACAC |
| pET28a-*pheDH*-check1 | ATGGAACACGAAGAAGTGAA |
| pET28a-*pheDH*-check2 | TTAGCTCAGACGGGCTGCAA |
